# Diet Practices, Body Mass Index, and Oral Health-Related Quality of Life in Adults with Periodontitis- A Case-Control Study

**DOI:** 10.3390/ijerph17072340

**Published:** 2020-03-30

**Authors:** Galit Almoznino, Naama Gal, Liran Levin, Eitan Mijiritsky, Guy Weinberg, Ron Lev, Avraham Zini, Riva Touger-Decker, Daniella Chebath-Taub, Boaz Shay

**Affiliations:** 1 Big Biomedical Data Research Laboratory, Hebrew University-Hadassah School of Dental Medicine, Jerusalem 91120, Israel; 2 Department of Oral Medicine, Sedation & Maxillofacial Imaging, Hebrew University-Hadassah School of Dental Medicine, Jerusalem 91120, Israel; 3 Department of Endodontics, Hebrew University-Hadassah School of Dental Medicine, Jerusalem 91120, Israel; dentidoc10@gmail.com (D.C.-T.); boaz@endo.co.il (B.S.); 4 In partial fulfillment of DMD degree, Hebrew University-Hadassah School of Dental Medicine, Jerusalem 91120, Israel; alonnaama@gmail.com; 5 Department of Periodontology, Faculty of Medicine and Dentistry, University of Alberta, Edmonton, AB T6G, Canada; liran@ualberta.ca; 6 Department of Otolaryngology, Head and Neck and Maxillofacial Surgery, Tel-Aviv Sourasky Medical Center, Sackler Faculty of Medicine, Tel Aviv 6139001, Israel; mijiritsky@bezeqint.net; 7 Department of Periodontology, Oral and Maxillofacial Center, Tel-Hashomer 5262000, Israel; guy.weinberg@gmail.com (G.W.); drronlev@gmail.com (R.L.); 8 Department of Community Dentistry, Hebrew University-Hadassah School of Dental Medicine, Jerusalem 91120, Israel; aviz@hadassah.org.il; 9 Rutgers School of Dental Medicine, Newark, NJ 07107, USA; decker@shp.rutgers.edu

**Keywords:** periodontitis, diet, body mass index (BMI), quality of life, oral health-related quality of life (OHRQoL)

## Abstract

*Objectives*: To assess and compare diet practices, body mass index (BMI), and oral health-related quality of life (OHRQoL) in adults with and without periodontitis. *Methods*: Demographics, health-related behaviors, BMI, dental and periodontal parameters, diet practices, and Oral Health Impact Profile-14 (OHIP-14) were collected from 62 periodontitis patients and 100 controls without periodontitis. *Results*: Having periodontitis was positively associated with male sex (*p* = 0.004), older age (*p* < 0.001), smoking pack-years (*p* = 0.006), weight (*p* = 0.008), BMI (*p* = 0.003), number of meals per day (*p* < 0.001) and had a negative association with decayed teeth (*p* = 0.013), alcohol (*p* = 0.006), and sweets (*p* = 0.007) consumption. Periodontitis patients were more likely to avoid carbonated beverages (*p* = 0.028), hot (*p* = 0.003), and cold drinks (*p* = 0.013), cold (*p* = 0.028), hard textured (*p* = 0.002), and fibrous foods (*p* = 0.02) than the controls, and exhibited higher global OHIP-14 (*p* < 0.001) and most domain scores. Age (*p* < 0.001), BMI (*p* =0.045), number of meals per day (*p* = 0.024), and global OHIP-14 score (*p* < 0.001) remained positively associated with periodontitis in the multivariate analysis. Conclusions: Periodontitis patients exhibited higher BMI and altered diet practices and OHRQoL as compared to controls. Assessment of diet practices, BMI, and OHRQoL should be part of periodontal work-up. Dentists and dietitians should collaborate to design strategies to address these challenges.

## 1. Introduction

There is a synergism between diet, nutrition, and the integrity of the oral cavity in health and illness [1]. Compromised oral health may impact functional abilities in regards to the ingestion of foods and fluids [2]. Over time, this can result in nutritional deficiencies and malnutrition which may, in turn, reduce resistance to infection and compromise tissue healing [3,4].

Periodontitis is a biofilm-induced chronic inflammatory disease that consists of loss of periodontal attachment, which eventually leads to tooth loss [5]. Assessing diet practices in patients with periodontitis is important for several reasons. First, tooth loss due to periodontitis may lead to an altered ability to bite and chew food. Prior research has revealed inverse associations between tooth loss and nutrient intake as well as diet quality [6,7,8]. Vice versa, poor nutrition such as reduced intake of carotin, vitamin A, C, dairy food, as well as vegetables were found to be correlated with loss of teeth [9].

Second, pain due to periodontitis may reduce appetite and subsequently alter the diet. Previous studies have shown that pain and discomfort in oral mucosa may negatively impact food intake and limit the types of food eaten [2,10].

A third reason to consider is the systemic impacts of nutrition and periodontal disease and their associations. Evidence of the effect of nutritional imbalance on cellular and molecular levels suggests that nutrition has the potential to affect biological gradient and thus affect periodontal infections [11]. Accordingly, periodontal diseases develop faster in undernourished populations [11]. Food intake is not a treatment for oral and dental diseases, but deficiencies of these nutrients can negatively impact tissue integrity, inflammation, bone mineralization, immune responses, and wound healing [12,13]. It is beyond the scope of this paper to review these mechanisms.

Oral health-related quality of life (OHRQoL) is a conceptual model that evaluates the functional, psychological, and social aspects and pain concerning oral and facial conditions [14]. An association exists between diet quality and better OHRQoL [15] and vice versa. We have previously demonstrated that OHRQoL was significantly impaired in patients with disorders of nutrition [16]. In the context of periodontitis, previous studies have shown that in patients with periodontitis, the OHRQoL is impaired compared with subjects without periodontitis [17,18,19].

To our knowledge, papers assessing the associations between having a diagnosis of periodontitis and diet practices, body mass index (BMI), and OHRQoL, while controlling for demographics, health-related habits, and dental and periodontal status, have not been published in the English language literature. This study hypothesized that there would be a correlation between the existence of periodontitis and higher BMI, altered diet practices and OHRQoL. To assess the hypothesis of the study, the research aimed to measure and compare BMI, diet practices, and OHRQoL between subjects with and without periodontitis who attended routine dental screenings and analyze the association between the existence of periodontitis and the following parameters: demographics, health-related behaviors, BMI, dental and periodontal parameters, diet practices, and OHRQoL.

## 2. Subjects and Methods

### 2.1. Study Population

The research was performed between January 1st, 2014 and June 30th, 2014, at the Department of Periodontology, Oral and Maxillofacial Center, Tel- Hashomer, Israel. The Oral and Maxillofacial facility is located at Sheba Hospital, which is the largest medical center in Israel and among the larger Middle East medical centers. The Department of Periodontology is a secondary dental facility that coordinates referrals nationwide from dozens of dental clinics of patients who need periodontal counseling and management. 

The periodontitis group was composed of 62 consecutive subjects that were diagnosed with periodontitis at the Department of Periodontology. The control group was composed of one hundred consecutive subjects who attended the Conservative Dentistry Department of this Oral and Maxillofacial Center, for elective dental screenings without a history of periodontitis (i.e., the same target population without the disease under investigation). All subjects were examined at their first visit before dental and/or periodontal treatment. At the completion of the examination, the subjects were provided with a written report detailing their dental and oral status and any diagnosed mucosal lesions, and this examination was recorded in their dental file. Patients were advised to seek consultation and treatment according to their findings.

WINPEPI softwarewas used to conduct sample size calculation [20] and showed that at least 147 subjects, composed of two groups that have a 40:60 ratio (92 subjects in one group and 55 subjects in the second group) were required to achieve eighty percent statistical power to distinguish a 4.48-point difference in Oral Health Impact Profile-14 (OHIP-14 global score, considering that α= 5%, and that the approximated standard deviations are 9.93 (of the group with highest standard deviation) and 8.25 (of the group with the lowest standard deviation),considering our previous publication assessing OHIP-14 [21].

Ethical issues: The research complies with the STrengthening the Reporting of OBservational studies in Epidemiology (STROBE) protocol. The study was approved by the Tel Hashomer institutional review board (approval number 1133-2011-version 3). All subjects signed informed consent before their participation in the research.

Eligibility criteria: To diminish confounding factors like aging and morbidity on periodontitis, diet practices as well as OHRQoL, the research solely included young to middle-aged participants without co-morbid physical disability and/or systemic disease (e.g., diabetes, cardiovascular diseases, malignant diseases, etc.) The reason for exclusion is that these diseases can increase the risk of inflammation and periodontitis as well as affect nutritional practices and the OHRQoL.

Inclusion criteria: new subjects attending elective dental examinations, age range: 18–55.

Exclusion criteria: urgent medical and/or dental/periodontal condition; disorders of nutrition; psychiatric conditions and/or using medications that affect mental state (e.g., antidepressants and benzodiazepines); drug, medications and/or alcohol abuse; physical disabilities; systemic diseases (e.g., diabetes, cardiovascular diseases, malignant diseases, etc.); immune-compromised status induced by chemotherapy, radiotherapy and/or chronic steroids use, pregnancy or lactation. The periodontitis group was diagnosed using the American Academy of Periodontology 1999 criteria [22,23]. Periodontal therapy six months before attendance was considered an exclusion criterion for the periodontitis group [24,25].

### 2.2. Collected Data

Questionnaires: The research included a self-administered questionnaire filled by the patients during the first appointment. Variables in the questionnaire were defined as follows:

Socio-demographic variables: (a) Age was presented in years; (b) Sex: male/ female;(c) Education was presented in years of schooling; (d) Country of the birth variable included: Africa, Asia, ‘West’ (North and South America, Europe, Australia), Former Soviet Union Israel and others, as described previously [16].

### 2.3. Health-Related Habits Variables

Smoking cigarettes, hookah and alcohol consumptions, and physical activity were described as yes/no questions. In addition, we performed a calculation of smoking pack-years by multiplying the number of packs smoked per day by the number of years of smoking [21]. Definitions of alcohol consumption habits and physical activity were described previously [21,26,27].

### 2.4. Oral Health-Related Quality of Life (OHRQoL).

The validated Hebrew version of the OHIP-14 [28] was used as a tool to measure OHRQoL [14]. OHIP-14 and domain scores were analyzed as continuous variables. The methods related to the application and analysis of the OHIP-14 score are described in detail in our prior published studies [16,21,29,30,31,32].

### 2.5. The DietPractices Questionnaire

Assessment of diet practices was done using a two-page modified food frequency questionnaire (FFQ) with additional questions assessing self-reported diet practices, which was developed by a U.S. Registered Dietitian (R.T.D.) and was subjected to content validation and reviewed by experts in nutrition practice and research [2] and has been used previously [2,16,33]. This questionnaire evaluated the frequency of consumption of the following food groups: fruits (fresh uncooked), salad or other raw, fresh vegetables, cooked vegetables, dairy products, carbohydrate-rich foods (e.g., bread, cereal, rolls, bagels), fish or meat, eggs, oil products, snacks (cracker, popcorn, bagel, nuts), sweets (croissant, chocolate, cookies, etc.), strawberries or other citrus fruit, tomatoes, fried foods [2,16]. The frequency of food group consumption was categorized on an ordinal scale as never, 1–2 servings/week, 3–4servings/week, 5–6servings/week, or daily [2,16,33]. The subjects were also asked to indicate if they avoid (yes/no) any of the following: sauces, seasonings, chewing gum, herbs, sparkling water, non-carbonated and carbonated beverages, and drinks they considered hot or cold, along with foods they considered hot, cold, hard, or fibrous texture. Subjects also indicated the number of meals per day, and whether they had followed a weight control diet in the past year [2,16,33].

### 2.6. Clinical Examination

All subjects underwent a clinical examination that included height, weight, and calculation of BMI as well as dental and periodontal examinations. The examiners (GW and DST) completed a training and calibration session to ensure mutual concurrence and appropriate interpretation of the indices measures employed in the research. All clinical examinations were performed on the subjects with periodontitis as well as on the controls.

Body Mass Index (BMI): Height, weight was measured and BMI was defined as the weight (in kilograms) in light clothing divided by height squared (in meters) [34]. BMI was analyzed as a continuous parameter.

Dental and periodontal examinations: were performed by the aid of light, a dental mirror, and a standard periodontal probe. Radiological evaluation incorporated vertical bilateral bitewings including the molar and premolar areas as well as parallel periapical radiographs of the incisors in the maxilla and mandible. Radiographs were performed in the same appointment as the clinical examination and the questionnaire. The following measurements were included:

1. DMFT index (Decay, Missing, and Filled Teeth) was used for caries status assessment, following the WHO criteria. The calculation included 28 teeth (third molars were excluded) (http://www2.paho.org/hq/dmdocuments/2009/OH_st_Esurv.pdf). Whilst the missing teeth (M) ingredient of the DMFT index contains teeth missing only due to caries, in adults, particularly in periodontitis, determination of the specific reason for tooth loss can be a challenge [17]. Therefore, the present study included missing teeth of any reason and was not limited to dental caries.

2. Plaque Index (PI): a measurement of oral hygiene by calculating the percentage of teeth with visible plaque presence on any tooth surface (third molars teeth excluded) [35].

3. Probing depth (PD) and bleeding on probing (BOP). The six Ramfjord index teeth (teeth number: 16, 21, 24, 36, 41, 44) and the remaining molar teeth 26, 46, were clinically tested. Ramfjord index teeth are well known as a representative of the different types of teeth [36]. Six points around the tooth were used for each index measurement [17,37,38]. For probing depth, the value of the highest measurement was recorded and the value for the bleeding score was calculated as the percent of bleeding pockets from the total pockets measured [37].

### 2.7. Statistical Analysis

Analysis of the data was performed with the SPSS software version 22.0 (Chicago, Illinois, United States) and statistical significance was defined as a p-value of less than 5 percent. Continuous variables were presented as means and standard deviations. The categorical variables were presented as percentages and frequencies.

Tests to assess associations and correlations between the groups’ parameter (periodontitis vs. controls) as a dependent variable and the independent variables included: Person Chi-square, Fisher’s exact test or likelihood ratio for categorical variables, as well as an analysis of variance (ANOVA) test for the numerical variables. Based on the univariant results, the significant independent parameters were selected for logistic regression analysis (with the groups’ parameter as a dependent variable).

## 3. Results

A total of 162 subjects (100 in the control group and 62 in the periodontitis group) participated in the study with a mean age of 26.77 ± 9.11 years. Table 1 presents the demographics and health-related habits of the study population. Compared to controls, those in the periodontitis group were significantly more likely to be male (*p* = 0.004), older (*p* < 0.001), and to have more smoking pack-years (*p* = 0.006). There was a negative association between the periodontitis group and alcohol consumption (*p* = 0.006). The periodontitis and the controls did not statistically significantly differ regarding birth country (*p* = 0.343), smoking cigarettes (*p* = 0.746) and hookah (*p* = 0.224), physical activity (*p* = 0.668), the performance of a weight control diet during the past year (*p* = 0.145), and years of schooling (*p* = 0.300) (Table 1).

Table 2 presents BMI, dental and periodontal parameters of the study population. Those in the periodontitis group were significantly more likely to be of a higher weight (*p* = 0.008) and BMI (*p* = 0.003), have more missing-M and (*p* < 0.001) filled teeth-F (*p* = 0.007), and higher DMFT scores (*p* = 0.006), probing depth (*p* < 0.001), and plaque index scores (*p* < 0.001). Being in the periodontitis group was negatively associated with having decayed teeth-D (*p* = 0.013). There were no significant differences between the periodontitis and the control groups regarding height (*p* = 0.103) and bleeding score (*p* = 0.912) (Table 2).

Table 3 presents the statistically significant differences in the diet practices between the two groups. Being in the periodontit is group was positively associated with the number of meals per day (*p* < 0.001) and negatively associated with the frequency of eating sweets (*p* = 0.007). Compared to controls, those in the periodontitis group were significantly more likely to avoid consumption of carbonated beverages (*p* = 0.028), and drinks they considered hot (*p* = 0.003)and cold (*p* = 0.013), and foods they considered cold (*p* = 0.028), hard textured(*p* = 0.002), and fibrous (*p* = 0.02) (Table 3). Overall, for all the questions of foods, one might avoid, a greater proportion of individuals with periodontitis were more likely to avoid specific types of fluids and foods than those without periodontitis.

There were no significant differences between the groups in the frequency of consumption of fruits (*p* = 0.762), salad or fresh vegetables (*p* = 0.651), cooked vegetables (*p* = 0.862), dairy (*p* = 0.142), carbohydrate rich foods(*p* = 0.252), fish or meat (*p* = 0.681), eggs (*p* = 0.667), oil products(*p* = 0.553), snacks (*p* = 0.220), strawberries (*p* = 0.143), citrus fruits (*p* = 0.133), tomatoes (*p* = 0.281), fried food (*p* = 0.861). Additionally, there were no statistically significant differences between the periodontitis and the control groups regarding avoidance of sauces (*p* = 1.00), seasonings (*p* = 1.00), herbs (*p* = 1.00), and chewing gum (*p* = 0.069).

Analysis of OHIP-14 domains and global scores of the study population are presented in Table 4. Compared to the control group, those in the periodontitis group exhibited significantly higher global OHIP (*p* < 0.001) and domain scores [except the social disability scores (*p* = 0.052, which is close to statistical significance)]. The highest score was noted in the physical pain domain, while the lowest mean score was noted in the functional limitation domain.

Multivariable logistic regression analysis was performed with the parameters that had significant associations/correlations with the periodontitis group compared to the control group in the univariate statistical analyses. Multivariate analysis revealed that compared to the control group, those in the periodontitis group were significantly more likely to eat more meals per day (*p* = 0.024,standard error [SE]: 0.41, odds ratio 2.54, 95% confidence interval [CI]:1.13–5.69), be older (*p* < 0.001, SE = 0.035, OR = 1.15, 95% CI: 1.07–1.23), have a higher global OHIP-14 score (*p* < 0.001, SE = 0.43, OR = 1.16, 95% CI:1.06–1.26), and have a higher BMI (*p* = 0.045, SE = 0.04, OR = 1.09, 95%CI 1.01–1.20). The Nagelkerke R square, which represents the proportion of the total variability that the model explains was 71%.

## 4. Discussion

To our knowledge, associations between diet practices and OHRQoL in adults with periodontitis in comparison to a control sample have not been published in the English literature. We explored these associations in addition to important potential confounding parameters such as demographics, health-related habits, BMI, and dental and periodontal status. The results of the current research confirmed our hypothesis that adult patients with periodontitis have altered diet practices and lower OHRQoL compared to individuals without periodontitis.

Associations between periodontitis and demographic characteristics, health-related habits, BMI, dental and periodontal parameters, and OHRQoL.

*Demographics characteristics and periodontitis.* Subjects with and without periodontitis did not statistically significantly differ regarding birth country and years of schooling. However, male sex and older age had a positive association with having a diagnosis of periodontitis as compared to not having it.

*Sex.* Robust evidence from systematic reviews and cross-sectional studies show that the prevalence of periodontitis is lower in females compared to males [39,40,41]. However, men do not seem to be at increased risk for more periodontal loss compared to women [39]. Instead, sex differences are attributed to poorer oral hygiene, less favorable attitudes toward oral health, and poor dental-visit behavior among males than to any genetic factors [42].

*Age.* Of all the demographic characteristics that were analyzed, only age retained a statistically significant positive correlation with periodontitis in the multivariate analysis. Consistent with our findings, the likelihood of developing the periodontal disease rises with age in populations all over the globe, which suggests that age is a significant risk indicator [43]. The new staging and grading framework of periodontitis classification states that age can increase the risk for future progression of alveolar bone loss [44]. Nevertheless, since both groups were not comparable in terms of age group, and since age retained a significant statistical association in the multivariate analysis, we may attribute some of the impacts on oral health and food intake to the age factor rather just periodontitis.

*Education.* Interestingly, in the present study, we found no statistical difference of positive and negative periodontitis related to years of education, in contrast to other studies that show that increased risk of periodontitis is associated with low level of education [45].

Our findings suggest that high risk behaviors such as alcohol consumption, smoking, and food consumption are influenced from hypersensitivities and pain related to periodontitis, rather than of higher education. Indeed, it had been previously revealed that there is an insufficient level of awareness of cardiovascular risk factors, even among medical students [46].

*Health-related habits and periodontitis.* The periodontitis group had a positive association with smoking pack-years and a negative association with alcohol consumption.

*Smoking.* Although smoking (yes/no) did not differ between the groups, smoking pack-years, indicative of both the amount and time of consumption, were positively associated with periodontitis. Consistent with this finding, there is accumulating evidence for a higher prevalence of periodontitis among smokers [43]. Smoking is considered the second most important risk factor (after inadequate plaque control) in the etiology and pathogenesis of periodontitis [47] and a grade modifier in the new classification [44].

*Alcohol.* Alcohol consumption among controls was higher compared to the periodontitis group. A possible explanation for the avoidance of alcohol could be dentinal hypersensitivity, consistent with reports of a higher rate of dentinal hypersensitivity in patients with periodontitis [48]. In an in-vitro study, it has been shown under an electron microscope scanning that red and white wine has an etching effect, which results in greater exposure of dentinal tubules and thus increases sensitivity [49]. Furthermore, alcohol has been found in literature as a condition that may worsen periodontitis [50].

*BMI and periodontitis*. Those in the periodontitis group had a higher BMI compared to controls. While both fell within the “normal weight” range as defined for BMI (18.5–24.9), the mean BMI was clinically and statistically significantly higher (24.3) in the periodontitis group compared to the controls (20.8). The definition of overweight is a BMI of 25.0–30.0 [51].Associations between obesity and periodontitis have been published [52,53], although the causal evidence is lacking [54]. The chronic inflammatory state and oxidative stress which lead to the development of insulin resistance may be involved in the association between obesity and periodontal disease [55].

*Dental and periodontal parameters and periodontitis*. Those in the periodontitis group had less tooth decay than those in the control group. Findings concerning the relationship between periodontitis and dental caries are contradictory. Prior research on this association has been heterogeneous, some has documented a positive association [56], however, recent research showed a negative correlation between the severity of periodontitis and caries prevalence [57], which is consistent with our findings. A possible explanation for the lower decay score among the periodontitis group (see Table 2), could be our finding of a lower consumption rate of sweets in the periodontitis group (see Table 3) that had been demonstrated in the study.

As expected, missing teeth, probing depth, and plaque index were found to be higher in the periodontitis group than in the control group. The higher number of missing teeth among patients with periodontitis (see Table 2) could account for the avoidance of certain foods. The majority of research regarding tooth loss and diet has been done with older adults and demonstrates that those adults with tooth loss consume significantly lower amounts of fresh fruits and vegetables than those with complete dentition [6]. Using the National Health and Nutrition Examination Survey (NHANES) data, Zhu and Hollis found that adults age 19 years of age and older with 20 or fewer teeth consumed lower quality diets and lower intakes of fruits, vegetables, and whole grains than adults with 21–27 teeth [6].

It is also important to assess plaque index as a possible confounder since the plaque index is directly affected by diet habits and can vary depending on types of foods consumed and oral hygiene practices [58]. The bleeding score could result from extensive carious lesions involving the gingiva and not necessarily from periodontitis, which can explain why this parameter was not significantly different between the groups.

*Diet practices and periodontitis*. Although those in the periodontitis group were more likely to eat 4 or more meals per day than those in the control group, this only represented 16.4% of the population and 42.5% only ate 1–2 meals per day. Those in the periodontitis group more often avoided alcohol, sweets, carbonated beverages, hot and cold drinks, cold food, hard textured and fibrous foods more often than controls (Table 3). These observations could result from the fact that periodontitis is associated with gingival recessions and a high rate of dentinal hypersensitivity as well as with a higher number of missing teeth and as a result, reduced chewing ability. For example, the periodontitis group consumed fewer sweets, with 11% of the control group who eat sweets daily compared to 0% in the periodontitis group (Table 3). High osmotic stimuli such as sugar can result in fluid flow within the dentinal tubules and cause painful sensations [48]. It is also known that cemental exposure can cause sensitization to cold drinks and food [59,60]. Some studies suggest that carbonated beverages are a risk factor for developing periodontitis [61] attributed to chronic inflammation, due to their high dietary glycemic load [61]. It had been suggested that the periodontal health of patients could benefit from lowering carbonated beverage consumption [62].

The avoidance of foods in the periodontitis group considered hard or fibrous may be related to the greater degree of tooth loss in this group as compared to the control group. Prior research has shown that individuals with tooth loss are more likely to avoid foods they consider hard or fibrous [8,63]. In particular, reduced chewing capacity and the discomfort that characterizes periodontitis can affect the patient’s diet [64]. The lack of significant differences in other food group intake, particularly for fruits and vegetables may be related to actual foods consumed. For example, in Israel, salads are often served chopped and the altered consistency may allow for positive adaptive behaviors in this regard. Further study in regards to specific foods with a larger sample is needed to explore these associations.

*Oral health-related quality of life (OHRQoL) and periodontitis*. Consistent with prior research [17,19], in the present study, those in the periodontitis group exhibited significantly worse global OHIP scores as well as in most domain scores than those in the control group. Periodontitis had been shown to have a wide range of clinical characteristics that may adversely affect the OHRQoL [65]. The impact of periodontitis can be expressed in many ways including social, physical, and psychological means [66]. Additionally, OHRQoL had been shown to deteriorate with the increase in the severity of periodontitis [67]. The loss of periodontal tissues and greater numbers of missing teeth in patients with periodontitis may account for the functional limitations and increase in the OHIP-14 scores. Specifically, questions 7 and 8 in the OHIP-14 questionnaire, representing the physical disability domain (see Table 4), that asks about inadequate diet and stopping a meal because of dental, mouth, or dentures problems, can reveal the connection between food avoidance and low OHRQoL. Pain and discomfort associated with periodontitis could impact the physical pain domain of the OHIP-14 score, and indeed the physical pain domain achieved the highest OHIP-14 domain scores, suggesting that pain has the highest contribution in lowering OHRQoL in these patients. Findings suggested that periodontitis patients avoid hard and fibrous food, which may be attributed more to the impact of physical pain (which showed the highest mean score among other OHIP domains), rather to the impact of the functional limitations (which showed the lowest mean score among other OHIP domains). Finally, the OHRQoL retained its statistically significant association with periodontitis in the multivariate analysis, which considered many potential confounders.

*Strengths and limitations*. Strengths of this study include the relatively large sample size (162 subjects) and the strict protocol that employed standardized internationally accepted questionnaires of the OHIP-14 and the structured modified food frequency questionnaire (FFQ), as well as clinical examinations for all subjects that included BMI as well as DMFT index (Decay, Missing, and Filled Teeth) Plaque Index (PI), Probing depth (PD) and bleeding on probing (BOP). This will enable a future studied comparison with other populations with different socioeconomic and ethnic status. We diminished confounding factors such as aging and morbidity on periodontitis, diet practices, and OHRQoL, by including only young to middle-aged subjects without co-existing physical disabilities and/or systemic diseases.

The limitations of the study include the convenience samples which may limit the generalizability of the results. Nevertheless, the samples were composed of patients who were referred from multiple clinics serving different populations. Moreover, since we used a case-control study design, we could not presume causal relations between the variables, and therefore there is only a description of associations and correlations between the variables. While we analyzed numerous parameters, considering the depth and complexities of the issues, other parameters may impact the results that were not considered. There is a need for further longitudinal studies, that will be based on the new classification of periodontal conditions [68] in other settings and populations and in more ranges of age that will include laboratory analysis of chemical elements and bone metabolism to address these issues.

## 5. Conclusions

In this sample of patients, those with periodontitis exhibited impaired diet practices, higher BMI values, and worse OHRQoL compared to controls. These findings highlight the need for oral health practitioners to consider the assessment of diet practices, BMI, and OHRQoL as an integral part of a periodontitis evaluation and treatment plan. Dentists should refer patients with altered diet habits to a dietitian for care. Dentists and dietitians should collaborate to design strategies to address these challenges.

## Figures and Tables

**Table 1:** Demographics and health-related habits of the study population.

**Parameter**		**Variable**	**Periodontitis**		**Control**		**Total**			***p*** **Value**
			**N**	**%**	**N**	**%**	**N**		**%**	
Sex		Male	52	83.9	63	63	115		71	**0.004** *
		Female	10	16.1	37	37	47		29	
Birth country		Africa	1	1.6	0	0	1		0.6	0.343 **
		Asia	0	0	1	1	1		0.6	
		West	2	3.2	3	3	5		3	
		FSU	7	11.3	12	12	19		11.7	
		Israel	49	79	82	82	131		80.9	
		Other	3	4.8	2	2	5		3	
		No	53	85.5	76	76	129		79.6	
Smoking Cigarettes		Yes	22	35.5	33	33	55		34	0.746 *
		No	40	64.5	67	67	107		66	
Smoking Hookah		Yes	11	17.7	26	26	37		22.8	0.224 *
		No	51	82.3	74	74	125		77.2	
Alcohol consumption		Yes	38	61.3	81	81	119		73.5	**0.006** *
		No	24	38.7	19	19	43		26.5	
Physical Activity		Yes	50	80.6	78	78	128		79	0.688 *
		No	12	19.4	22	22	34		21	
Performed a weight control diet during the past year		Yes	9	14.5	24	24	33		20.4	0.145 *
		No	53	85.5	76	76	129		79.6	
**Parameter**		**N**	**Mean ± SD**	**95% Confidence Interval of Mean**						***p*** **Value**
				**Mean Lower Bound**				**Mean Upper Bound**		
Age	Control	100	23.27 ± 5.85	22.11				24.43		<**0.001** ^
	Periodontitis	62	32.42 ± 10.54	29.74				35.10		
	Total	162	26.77 ± 9.11	25.36				28.19		
Years of schooling	Control	100	13.15 ± 1.93	12.77				13.53		0.300 ^
	Periodontitis	60	13.52 ± 2.49	12.87				14.16		
	Total	160	13.29 ± 2.15	12.95				13.62		
Smoking Pack Years	Control	100	0.9 ± 2.8	0.4				1.5		**0.006** ^
	Periodontitis	62	3.0 ± 6.4	1.4				4.6		
	Total	161	1.7 ± 4.6	1.0				2.5		

*Pearson Chi-square; ** likelihood ratio; ^ ANOVA.

**Table 2:** Anthropometrics, dental and periodontal parameters of the study population.

Parameters	Variable		N	Mean ± SD	95% Confidence Interval for Mean		*p* Value *
					Lower Bound	Upper Bound	
Anthropometric	Height(meter)	Control	100	1.72 ± 0.09	1.70	1.74	0.103
		Periodontitis	62	1.74 ± 0.08	1.72	1.77	
		Total	161	1.73 ± 0.09	1.71	1.74	
	Weight (kg)	Control	100	69.3 ± 12.5	66.8	71.8	**0.008**
		Periodontitis	61	74.6 ± 11.5	71.6	77.6	
		Total	161	71.3 ± 12.4	69.4	73.2	
	BMI	Control	100	20. 8 ± 8.9	19.0	22.5	**0.003**
		Periodontitis	61	24.3 ± 3.1	23.5	25.1	
		Total	161	22.1 ± 7.5	20.9	23.3	
Dental and periodontal parameters	Decayed teeth (D)	Control	100	1.47 ± 2.30	1.01	1.93	**0.013**
		Periodontitis	62	0.65 ± 1.46	0.27	1.02	
		Total	162	1.15 ± 2.05	0.84	1.47	
	Missing teeth (M)	Control	100	0.16 ± 0.56	0.05	0.27	<**0.001**
		Periodontitis	62	1.15 ± 2.07	0.62	1.67	
		Total	162	0.54 ± 1.43	0.31	0.76	
	Filled teeth (F)	Control	100	4.90 ± 4.96	3.92	5.88	**0.007**
		Periodontitis	62	7.32 ± 6.12	5.77	8.88	
		Total	162	5.83 ± 5.54	4.97	6.69	
	DMFT	Control	100	6.21 ± 6.27	4.97	7.45	**0.006**
		Periodontitis	62	9.11 ± 6.64	7.43	10.80	
		Total	162	7.32 ± 6.55	6.30	8.34	
	Bleeding score	Control	100	31.47 ± 19.41	27.62	35.332	0.912
		Periodontitis	62	31.13 ± 19.46	26.18	36.074	
		Total	162	31.34 ± 19.37	28.33	34.352	
	Probing Depth	Control	100	3.00 ± 0.54	2.90	3.1181	<**0.001**
		Periodontitis	62	4.78 ± 1.02	4.52	5.0494	
		Total	162	3.69 ± 1.15	3.51	3.8699	
	Plaque index	Control	100	39.94 ± 26.90	34.60	45.28	<**0.001**
		Periodontitis	62	70.84 ± 26.23	64.18	77.50	
		Total	162	51.77 ± 30.54	47.03	56.51	

* ANOVA. The bold is statistically significant result.

**Table 3:** Frequency of consumption and avoidance patterns of specific food groups among the study population * Pearson Chi-square, ^ likelihood ratio ** Fisher’s exact test.

**Variable**	**Frequency of Consumption**	**Control**		**Periodontitis**			**Total**		***p*** **Value**
		**N**	**%**	**N**		**%**	**N**	**%**	
Meals/Day	1	2	2	2		3.2	4	2.5	<0.001 ^
	2	29	29	24		39.3	53	32.9	
	3	69	69	25		41	94	58.4	
	4<	0	0	10		16.4	10	6.2	
Sweet^^^/week	0	9	9	5		8.1	14	8.6	0.007 *
	1–2	32	32	35		56.5	67	41.4	
	3–4	32	32	13		21	45	27.8	
	5–6	16	16	9		14.5	25	15.4	
	Daily	11	11	0		0	11	6.8	
**Variable**	**Do you avoid the foods?**	**Control**		**Periodontitis**			**Total**		***p*-value** *
		**N**	**%**	**N**	**%**		**N**	**%**	
Carbonated beverage	Yes	2	2	7		11.3	9	5.6	0.028 **
	No	98	98	55		88.7	153	94.4	
Hot drinks	Yes	2	2	7		11.3	9	5.6	0.028 **
	No	98	98	55		88.7	153	94.4	
Cold drinks	Yes	0	0	6		9.7	6	3.7	0.003 **
	No	100	100	56		90.3	156	96.3	
Cold food	Yes	1	1	6		9.7	7	4.3	0.013 **
	No	99	99	56		90.3	155	95.7	
Hard texture food	Yes	2	2	6		9.7	8	4.9	0.028 *
	No	98	98	56		90.3	154	95.1	
Fibrous food	Yes	1	1	8		12.9	9	5.6	0.002 **

**Table 4:** The Mean Oral Health Impact Profile Scores (OHIP-14) global and domain scores of the study population.

		N	Mean ± SD	95% Confidence Interval for Mean		* *p* between Groups
				Lower Bound	Upper Bound	
OHIP 1+2Functional limitation	Control	100	0.1±0.3	0.05	0.2	**0.018**
	Periodontitis	62	0.3±0.6	0.1	0.4	
	Total	162	0.2±0.4	0.1	0.2	
OHIP 3+4 Physical pain	Control	100	0.6±0.6	0.5	0.7	<**0.001**
	Periodontitis	62	1.1±1.0	0.9	1.4	
	Total	162	0.8±0.9	0.7	0.9	
OHIP 5+6Psychological discomfort	Control	100	0.4±0.7	0.3	0.5	**0.021**
	Periodontitis	62	0.70±0.92	0.5	0.9	
	Total	162	0.51±0.79	0.4	0.6	
OHIP 7+8 Physical disability	Control	100	0.2±0.4	0.1	0.3	**0.013**
	Periodontitis	62	0.5±0.8	0.3	0.7	
	Total	162	0.3±0.6	0.2	0.4	
OHIP 9+10 Psychological disability	Control	100	0.3±0.5	0.2	0.4	<**0.001**
	Periodontitis	62	0.8 ±0.9	0.5	1.0	
	Total	162	0.5 ±0.7	0.3	0.6	
OHIP 11+12 Social disability	Control	100	0.2±0.4	0.1	0.3	0.052
	Periodontitis	62	0.4±0.7	0.2	0.6	
	Total	162	0.3±0.5	0.2	0.4	
OHIP 13+14 Handicap	Control	100	0.1±0.3	0.1	0.2	<**0.001**
	Periodontitis	62	0.4±0.7	0.3	0.6	
	Total	162	0.3±0.5	0.2	0.3	
OHIP-14 global score	Control	100	4.0±4.6	3.1	4.9	<**0.001**
	Periodontitis	62	8.6 ±9.5	6.2	11.0	
	Total	161	5.8±7.2	4.6	6.9	

*ANOVA. The bold is statistically significant result.

## List of Abbreviations

BOP	Bleeding upon probing
BMI	body mass index
DMFT	Decay, Missing, and Filled Teeth
OHIP-14	Oral Health Impact Profile-14
OHRQoL	Oral health-related quality of life
PI	Plaque index
PD	Probing depth
